# Hydraulic resistance of three-dimensional pial perivascular spaces in the brain

**DOI:** 10.1186/s12987-023-00505-5

**Published:** 2024-01-11

**Authors:** Kimberly A. S. Boster, Jiatong Sun, Jessica K. Shang, Douglas H. Kelley, John H. Thomas

**Affiliations:** Department of Mechanical Engineering, University of Rochester, Rochester, NY 14627 USA

**Keywords:** Perivascular spaces, Cerebrospinal fluid, Hydraulic resistance, Brain clearance system, Fluid dynamics, Hydraulic network models

## Abstract

**Background:**

Perivascular spaces (PVSs) carry cerebrospinal fluid (CSF) around the brain, facilitating healthy waste clearance. Measuring those flows in vivo is difficult, and often impossible, because PVSs are small, so accurate modeling is essential for understanding brain clearance. The most important parameter for modeling flow in a PVS is its hydraulic resistance, defined as the ratio of pressure drop to volume flow rate, which depends on its size and shape. In particular, the local resistance per unit length varies along a PVS and depends on variations in the local cross section.

**Methods:**

Using segmented, three-dimensional images of pial PVSs in mice, we performed fluid dynamical simulations to calculate the resistance per unit length. We applied extended lubrication theory to elucidate the difference between the calculated resistance and the expected resistance assuming a uniform flow. We tested four different approximation methods, and a novel correction factor to determine how to accurately estimate resistance per unit length with low computational cost. To assess the impact of assuming unidirectional flow, we also considered a circular duct whose cross-sectional area varied sinusoidally along its length.

**Results:**

We found that modeling a PVS as a series of short ducts with uniform flow, and numerically solving for the flow in each, yields good resistance estimates at low cost. If the second derivative of area with respect to axial location is less than 2, error is typically less than 15%, and can be reduced further with our correction factor. To make estimates with even lower cost, we found that instead of solving for the resistance numerically, the well-known resistance of a circular duct could be scaled by a shape factor. As long as the aspect ratio of the cross section was less than 0.7, the additional error was less than 10%.

**Conclusions:**

Neglecting off-axis velocity components underestimates the average resistance, but the error can be reduced with a simple correction factor. These results could increase the accuracy of future models of brain-wide and local CSF flow, enabling better prediction of clearance, for example, as it varies with age, brain state, and pathological conditions.

## Introduction

Perivascular spaces (PVSs) are annular channels that surround arteries and veins in the brain and are filled with cerebrospinal fluid (CSF). The flow of CSF along these PVSs is an important component of the brain’s glymphatic system, which distributes nutrients and removes metabolic waste products [1]. (See the recent reviews [2, 3]). The flow of CSF in this system can be usefully modeled as flow in a hydraulic network, with one-dimensional flows in individual components determined by their hydraulic resistance [4–9]. It is known that the hydraulic resistance of a PVS depends strongly on its size and shape [10–12]. In this paper we develop methods of estimating the hydraulic resistance of individual PVSs in the brain based on their known geometrical configuration, as determined from *in vivo* experimental data. The PVSs are often quite irregular in shape, and therefore it is useful to have ways of estimating their hydraulic resistance without doing a full numerical simulation of the detailed flow field.

Here we are concerned primarily with PVSs that can be considered as essentially open spaces, for which the flow is governed by the Navier-Stokes equation. PVSs surrounding pial (surface) arteries in the mouse brain are known to be essentially open spaces, unobstructed by tissue [13]. Far less is known about the amount of obstruction in PVSs surrounding penetrating arteries: experiments indicate that these PVSs in the mouse brain contain some mesh-like obstructions [14], but the blood vessel usually lies to one side of the PVS, an arrangement that usefully reduces the hydraulic resistance of the PVS only if it were an essentially open space [10]. It is likely that some PVSs in the brain contain a significant amount of tissue and might therefore be considered to contain a porous medium, with flow governed by the Darcy equation. Such might be the case for PVSs around arterioles and precapillaries deep in the brain. We present here (in Appendix 1) a method of estimating the hydraulic resistance of a porous PVS based on its detailed configuration, but since we know very little about this configuration, we do not carry out any specific applications of the method.

In a steady, laminar flow of fluid along an open duct, the volume flow rate $Q = \Delta p/R$ is proportional to the pressure drop $\Delta p$ between the entrance and exit of the duct and inversely proportional to a *hydraulic resistance* *R*, which can be calculated from the viscosity of the fluid and the detailed shape and length of the duct. Hydraulic resistance is analogous to electrical resistance, which impedes an electrical current (analogous to *Q*) driven by a given voltage drop (analogous to $\Delta p$). For nonuniform ducts, a more useful quantity is the *hydraulic resistance* $\mathcal {R}$ *per unit length*,

1 $$\begin{aligned} \mathcal {R} \equiv -\frac{\partial p/\partial z}{Q} , \end{aligned}$$

where $\partial p/\partial z$ is the pressure gradient in the direction of the flow (the *z*-direction). Simple geometric models of the cross section of open PVSs have been used to calculate their hydraulic resistance [10, 12], assuming that the cross section remains uniform along the PVS. Here we are seeking more accurate methods that account for the variations in the shape and cross-sectional area of a PVS, and hence its hydraulic resistance $\mathcal {R}$ per unit length, along its length.

**Fig. 1:**
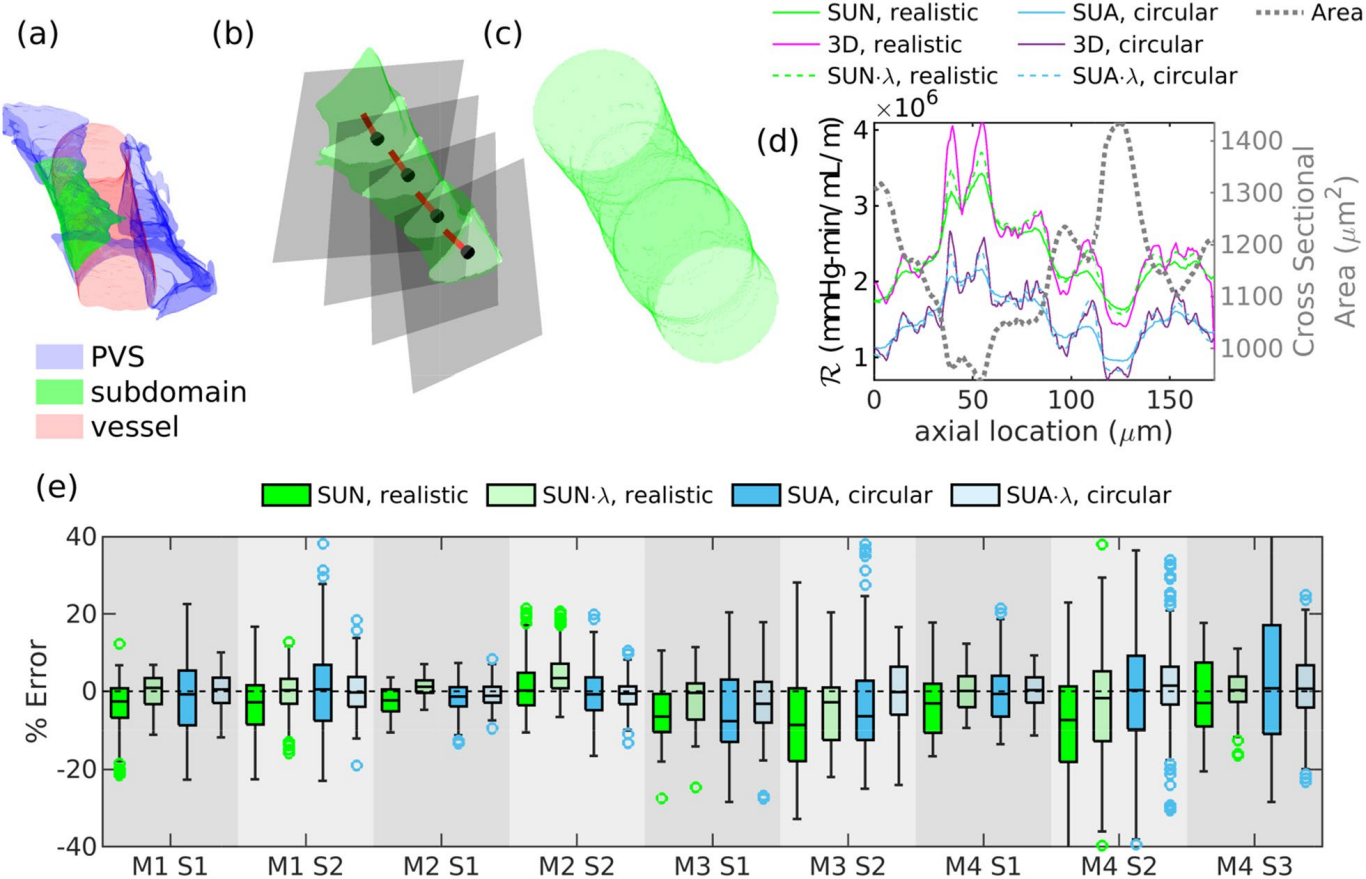
**a** Three-dimensional (3D) perivascular space (blue) and blood vessel (red) with an example subdomain (M1 S2, shown in green). **b** The subdomain with a few cross sections (gray) and corresponding center points (black) and axial vectors (red). **c** Circular duct with the same axial variation in cross-sectional area as the realistic geometry shown in (**b**). **d** Hydraulic resistance per unit length at cross sections along the length of the perivascular space and duct as calculated from the 3D simulations (“3D”), from the series unidirectional approach (“SUN” where calculated numerically; “SUA” where calculated analytically), and with a correction factor ($\lambda$ correction) to the series unidirectional approach (“SUN$\cdot \lambda$” and “SUA$\cdot \lambda$”), for both the perivascular space shown in (**b**) (“realistic”) and the circular duct shown in (c) (“circular”). **e** Error between the series unidirectional approximation and the 3D solution, for all cross sections. The box and whiskers plots indicate the median with a solid line and the interquartile range with a box. Outliers (points more than 1.5 times the interquartile range above the median) are shown with markers. The series unidirectional approximation underestimates the error, on average, but the correction factor reduces the error by more than half in most cases

## Pial PVS shapes determined from in vivo experimental data

We acquired and segmented three-dimensional (3D) two-photon microscopy images of murine pial PVSs, using the methods we developed and employed previously [12, 15]. We analyzed PVSs adjacent to pial arteries in four mice, M1–M4, and we considered two or three subdomains from each mouse, denoted as S1, S2, or S3, for a total of 9 different 3D segments of pial arteries. All of the subdomains were from pial PVSs located on the main branch of the MCA, between 4-7 bifurcations distal to the start of the MCA. Pial perivascular spaces are often shaped like incomplete annuli, with a lobe on each side of the pial artery but no spaces connecting the two lobes, as described by Raicevic et al. [12] and shown in Fig. 1a. All 9 of the segments we consider are shaped this way, and each is an individual lobe, located to one side of the pial vessel. Thus, the blood vessel is located to one side of the subdomains we consider here.

For each 3D configuration, we chose a series of points distributed along the vessel centerline, at each point finding the axial (centerline) direction and a cross section normal to that direction, as described in [15] and shown here in Fig. 1b. The normal cross sections are spaced approximately 0.7 µm apart, but the exact distance varies. Below, we will use a variety of methods to estimate the local value of $\mathcal {R}$ at each cross section.

For comparison, we created ducts with circular cross sections that have the same axial variation in cross-sectional area as the realistic geometries, as shown in Fig. 1c. This allows us to separate the effects of size and shape on $\mathcal {R}$. We do this by finding the cross-sectional area of the PVS at each of the previously-chosen normal cross sections, obtaining the area as a function of distance in the axial direction. We then interpolate the area function at 0.7 µm (1 pixel) intervals and create a duct with a straight centerline and circular cross sections of varying area, where the area as a function of axial distance is very nearly the same as in the original PVS geometry.

## Three-dimensional flow calculations for realistic PVS configurations

As a basis for testing the various simpler methods of approximating the hydraulic resistance of PVSs presented in the next section, we calculated the full 3D flow field for pressure-driven, laminar viscous flow in the actual observed PVS sections described above, and then calculated the hydraulic resistance for these flow fields. We solved the 3D Navier-Stokes equations using NX Flow in the Siemens NX Advanced Simulation software. The PVS geometries were meshed into 3D tetrahedral elements using NX advanced FEM. We used the fluid properties of water at $37^\circ$C. The parallel Flow Solver Scheme was set as the fully coupled Pressure-Velocity type, and we used a parallel solver to increase efficiency. From NX, we exported the results in CSV files that were then further post-processed in MATLAB.

We prescribed a zero-pressure condition at the outlet and no-slip conditions at the walls. In order to ensure fully-developed flow at the entrance of the PVS segment, we added a uniform (constant cross section) duct upstream whose cross-sectional shape matched the entrance of the segment and whose length was 40 µm. For the low Reynolds number flows considered here, the minimum length $z_L$ required to achieve a fully-developed velocity profile is $z_L \approx 0.5 D_h$, where $D_h$ is the *hydraulic diameter*, $D_h \equiv 4A/P$, where *A* is the cross-sectional area and *P* is the wetted perimeter of the duct [16]. In all cases we consider, $z_L \le 40$ µm. We prescribed a steady flow at the entrance of the inlet duct with a volume flow rate of *Q* = 2.19$\times 10^{4}$ µm$^3$/s, a typical value for pial PVSs in the mouse brain [15].

To ensure the accuracy of the 3D flow calculations, we reduced the mesh size in a finite-element model of a uniform (constant cross section) duct with circular cross section until the resistance was within 0.5% of that given by the exact analytical solution for laminar flow in a circular duct (Hagen-Poiseuille flow). This occurred for a mesh size of 0.5 µm. We determined that further reducing the mesh size to 0.3 µm resulted in a change in average resistance of less than 0.2%. We then used 0.3 µm for all the simulations, although it is clear from these grid verification studies that a coarser mesh would have produced very similar results.

We used a nonuniform mesh, in which the mesh elements were finer near the boundaries and coarser near the center. This varying mesh size was dictated by changing the internal gradation rate, which limits size differences among adjacent mesh elements, particularly in the radial direction (inward from the boundary). In order to verify that this did not affect the results, we ran a simulation in which we kept the mesh size at 0.5 µm while decreasing the internal graduation rate from 1.05 to 1.01, which resulted in a difference in average resistance of only 0.03%. We then used an internal gradation rate of 1.05 for all the simulations.

We calculated the hydraulic resistance per unit length at each normal cross section along the length of the channel by taking the volume-weighted average of the pressure at all elements in 2-µm-thick slices and dividing the pressure difference between adjacent slices by the volume flow rate *Q*. (See Appendix 3 for a discussion of how this calculated quantity compares to the theoretical one derived from the lubrication approximation in §4.1.) We observed an increase in error in cross sections near the outlet due to exit effects, so in all of our results we exclude the last five normal cross sections.

## Approximate methods for calculating the hydraulic resistance

### The lubrication approximation

Perivascular flows are generally characterized by very low Reynolds number (Re $\ll 1$), where viscous effects greatly outweigh inertial effects. Also, the pulsatile flows in PVSs have very small Womersley number, and hence the hydraulic resistance experienced by these flows is the same as that for a steady flow [17]: therefore, without loss of generality, we will consider steady flow throughout this paper. The Navier-Stokes equation can then be reduced to the Stokes (creeping flow) equation for steady flow,

2 $$\begin{aligned} 0 = -{\varvec{\nabla }}p + \mu \nabla ^2 {\textbf {u}}, \end{aligned}$$

where ${\textbf {u}}= (u_x, u_y, u_z)$ is the Eulerian velocity in Cartesian coordinates (*x*, *y*, *z*), *p* is the pressure, and $\mu$ is the dynamic viscosity. This equation is accompanied by the continuity equation for incompressibe flow,

3 $$\begin{aligned} {\varvec{\nabla }}\cdot {\textbf {u}}= 0 . \end{aligned}$$

Because PVSs are generally much longer (in the flow direction) than they are wide (in directions transverse to flow), the flow can be modeled using lubrication theory, which describes a class of flows that are nearly unidirectional. Even if the PVS changes in size or shape along its length, the magnitude of the axial flow is much larger than the transverse flow.

We can arrive at the lubrication equations for a duct by non-dimensionalizing the components of the Stokes equation according to:

4 $$\begin{aligned} {\hat{x}}=\, & {} \frac{x}{\sqrt{b_{0} c_{0}}}, \quad {\hat{y}}=\frac{y}{\sqrt{b_{0} c_{0}}}, \quad {\hat{z}}=\frac{z}{L}, \end{aligned}$$

5 $$\begin{aligned} {\hat{u}}=\, & {} \frac{u_{x}}{u_{c}}, \quad {\hat{v}}=\frac{u_{y}}{v_{c}}, \quad {\hat{w}}=\frac{u_{z}}{w_{c}}, \end{aligned}$$

6 $$\begin{aligned} {\hat{p}}=\, & {} \frac{p}{\Delta p_{c}}, \end{aligned}$$

where $b_0$ and $c_0$ are the characteristic length scales in the *x* and *y* directions (transverse to flow), respectively, *L* is the characteristic length scale in the *z* direction (axial), $u_c$, $v_c$, and $w_c$ are the characteristic velocities in the *x*, *y*, *z* directions, respectively, and $\Delta p_c$ is a characteristic pressure drop across the full length of the duct. We assume here that $b_0$ and $c_0$ are comparable, such that there is one characteristic length in the transverse direction, $\sqrt{b_0 c_0}$, and hence $u_x$ and $u_y$ are comparable, such that $u_c = v_c$. By non-dimensionalizing the continuity equation

7 $$\begin{aligned} \frac{\partial u_x}{\partial x}+\frac{\partial u_{y}}{\partial y}+\frac{\partial u_{z}}{\partial z}=0 , \end{aligned}$$

we find that $u_c = \alpha w_c$ where $\alpha \equiv \frac{\sqrt{b_0c_0}}{L}$ is the aspect ratio of the channel, and that the characteristic pressure is $\Delta p_c = \mu w_c L/b_0 c_0$.

The resulting dimensionless equations are:

8 $$\begin{aligned} 0=\, & {} \frac{\partial {\hat{u}}}{\partial {\hat{x}}}+\frac{\partial {\hat{v}}}{\partial {\hat{y}}}+\frac{\partial {\hat{w}}}{\partial {\hat{z}}}, \end{aligned}$$

9 $$\begin{aligned} 0=\, & {} -\frac{\partial {\hat{p}}}{\partial {\hat{x}}} + \alpha ^2 \left( \frac{\partial ^2}{\partial {\hat{x}}^2} + \frac{\partial ^2}{\partial {\hat{y}}^2} + \alpha ^2 \frac{\partial ^2}{\partial {\hat{z}}^2}\right) {\hat{u}}, \end{aligned}$$

10 $$\begin{aligned} 0= & {} -\frac{\partial {\hat{p}}}{\partial {\hat{y}}} + \alpha ^2 \left( \frac{\partial ^2}{\partial {\hat{x}}^2} + \frac{\partial ^2}{\partial {\hat{y}}^2} + \alpha ^2 \frac{\partial ^2}{\partial {\hat{z}}^2}\right) {\hat{v}}, \end{aligned}$$

11 $$\begin{aligned} 0= & {} -\frac{\partial {\hat{p}}}{\partial {\hat{z}}} + \left( \frac{\partial ^2}{\partial {\hat{x}}^2} + \frac{\partial ^2}{\partial {\hat{y}}^2} + \alpha ^2 \frac{\partial ^2}{\partial {\hat{z}}^2}\right) {\hat{w}}. \end{aligned}$$

We can see that several terms in these equations are small perturbations when $\alpha ^2 \ll 1$, as is the case for long, narrow ducts like PVSs. Hence we can appropriately express the variables as power series in $\alpha ^2$:

12 $$\begin{aligned} {\hat{p}}=\, & {} {\hat{p}}_0 + \alpha ^2 {\hat{p}}_2 + \alpha ^4 {\hat{p}}_4 + O(\alpha ^6), \end{aligned}$$

13 $$\begin{aligned} {\hat{u}}=\, & {} {\hat{u}}_0 + \alpha ^2 {\hat{u}}_2 + \alpha ^4 {\hat{u}}_4 + O(\alpha ^6), \end{aligned}$$

14 $$\begin{aligned} {\hat{v}}=\, & {} {\hat{v}}_0 + \alpha ^2 {\hat{v}}_2 + \alpha ^4 {\hat{v}}_4 + O(\alpha ^6), \end{aligned}$$

15 $$\begin{aligned} {\hat{w}}=\, & {} {\hat{w}}_0 + \alpha ^2 {\hat{w}}_2 + \alpha ^4 {\hat{w}}_4 + O(\alpha ^6). \end{aligned}$$

Substituting these series expressions into the governing equations and collecting terms of the same order of $\alpha ^2$, we find that the $0^{\mathrm{th}}$-order equations (composed of terms free of $\alpha ^2$) are

16 $$\begin{aligned} 0=\, & {} \frac{\partial \hat{u}_0}{\partial {\hat{x}}}+\frac{\partial \hat{v}_0}{\partial {\hat{y}}}+\frac{\partial \hat{w}_0}{\partial {\hat{z}}}, \end{aligned}$$

17 $$\begin{aligned} 0=\, & {} \frac{\partial \hat{p}_0}{\partial {\hat{x}}} = \frac{\partial \hat{p}_0}{\partial {\hat{y}}}, \end{aligned}$$

18 $$\begin{aligned} 0=\, & {} -\frac{\partial \hat{p}_0}{\partial {\hat{z}}} + \left( \frac{\partial ^2}{\partial {\hat{x}}^2} + \frac{\partial ^2}{\partial {\hat{y}}^2}\right) {\hat{w}}_0 . \end{aligned}$$

These $0^{\mathrm{th}}$-order equations constitute “classical” lubrication theory. At this order, pressure does not vary in the transverse direction, as the equations show. We later approximate the PVS as a series of sections, and solve these equations for each section, referring to the combined result as the “series unidirectional approximation”.

We find the $2^{\mathrm{nd}}$-order equations to be:

19 $$\begin{aligned} 0=\, & {} \frac{\partial {\hat{u}}_2}{\partial {\hat{x}}}+\frac{\partial {\hat{v}}_2}{\partial {\hat{y}}}+\frac{\partial {\hat{w}}_2}{\partial {\hat{z}}}, \end{aligned}$$

20 $$0 = {\mkern 1mu} {\text{ }} - \frac{{\partial \hat{p}_{2} }}{{\partial \hat{x}}} + \left( {\frac{{\partial ^{2} }}{{\partial \hat{x}^{2} }} + \frac{{\partial ^{2} }}{{\partial \hat{y}^{2} }}} \right)\hat{u}_{0} ,$$

21 $$\begin{aligned} 0=\, & {} -\frac{\partial {\hat{p}}_2}{\partial {\hat{y}}} + \left( \frac{\partial ^2}{\partial {\hat{x}}^2} + \frac{\partial ^2}{\partial {\hat{y}}^2}\right) {\hat{v}}_0, \end{aligned}$$

22 $$\begin{aligned} 0=\, & {} -\frac{\partial {\hat{p}}_2}{\partial {\hat{z}}} + \left( \frac{\partial ^2}{\partial {\hat{x}}^2} + \frac{\partial ^2}{\partial {\hat{y}}^2}\right) {\hat{w}}_2+\frac{\partial ^2 {\hat{w}}_0}{\partial {\hat{z}}^2}. \end{aligned}$$

The second-order (and higher-order) corrections constitute “extended” lubrication theory, where pressure does vary in the transverse direction, and, most critically for this study, depends on spatial variations of the duct cross section. Extended lubrication theory has been applied to two-dimensional channels, circular ducts, and elliptical ducts, and shown to be quite effective at capturing the effects of axial variations of the cross section when compared with experiments and full numerical simulations [18–20].

#### A nonuniform elliptical duct

In this section, we apply the lubrication model to solve for the flow in a nonuniform elliptical duct, whose varying cross section is an ellipse with semi-major and semi-minor axes *b*(*z*) and *c*(*z*):

23 $$\begin{aligned} \left( \frac{x}{b(z)}\right) ^2+\left( \frac{y}{c(z)}\right) ^2 = 1. \end{aligned}$$

While an actual PVS does not have elliptical cross sections, the nonuniform elliptical duct is a general shape for which we can find analytical expressions for the velocity and pressure at the $0^{\mathrm{th}}$ and $2^{\mathrm{nd}}$ orders, and which illustrates the effect that axial variations in cross-sectional shape and area can have on the resistance per unit length beyond what is given by classical lubrication theory. Though we arrived at dimensionless forms of the 0th and 2nd order equations in the previous section, to calculate resistances and compare them with those obtained with numerical solutions, we will solve the equations in dimensional form in the subsequent sections.

**Uniform duct approximation (**$0^{\mathrm{th}}$**-order solution).** The $0^{\mathrm{th}}$-order, dimensional lubrication equations are:

24 $$\begin{aligned} 0=\, & {} \frac{\partial {u_0}}{\partial {x}}+\frac{\partial {v_0}}{\partial {y}}+\frac{\partial {w_0}}{\partial {z}}, \end{aligned}$$

25 $$\begin{aligned} 0=\, & {} \frac{\partial {p_0}}{\partial {x}} = \frac{\partial {p_0}}{\partial {y}}, \end{aligned}$$

26 $$\begin{aligned} 0=\, & {} -\frac{d{p_0}}{d {z}} + \mu \left( \frac{\partial ^2}{\partial {x}^2} + \frac{\partial ^2}{\partial {y}^2}\right) {w}_0. \end{aligned}$$

We see that the *z*-momentum equation takes the form of the Poisson equation, and hence the axial velocity $w_0(x,y,z)$ can be readily obtained numerically for a duct of arbitrary cross section. For an elliptical duct, by enforcing a no-slip condition on the boundary, the axial velocity can be obtained analytically:

27 $$\begin{aligned} w_0 =\, \frac{1}{2\mu }\frac{dp_0}{dz}\frac{b^2c^2}{b^2+c^2}\left( \left( \frac{x}{b}\right) ^2+\left( \frac{y}{c}\right) ^2-1\right) . \end{aligned}$$

The flow rate *Q* is independent of *z*, and we can determine the pressure gradient in the duct by applying an integral constraint

28 $$\begin{aligned} Q=\, & {} 4 \int _{0}^{c} \int _{0}^{b \sqrt{1-(y / c)^{2}}} w \ dx \ dy \end{aligned}$$

29 $$\begin{aligned}=\, & {} 4 \int _{0}^{c} \int _{0}^{b \sqrt{1-(y / c)^{2}}} (w_0+\alpha ^2 w_2 + O(\alpha ^4)) \ dx \ dy . \end{aligned}$$

To close the problem, we assume that the bulk of *Q* is determined only by the $0^{\mathrm{th}}$ order flux:

30 $$\begin{aligned} Q\approx 4 \int _{0}^{c} \int _{0}^{b \sqrt{1-(y / c)^{2}}} w_0 \ dx \ dy; \end{aligned}$$

the justification for this assumption is given in Appendix 2. Using this integral constraint, we can determine the pressure gradient,

31 $$\begin{aligned} \frac{d p_{0}}{d z}=-\frac{4 \mu Q}{\pi } \frac{b^{2}+c^{2}}{b^{3} c^{3}}. \end{aligned}$$

The $0^{\mathrm{th}}$ order hydraulic resistance per unit length is then

32 $$\begin{aligned} \mathcal {R}_0 = \frac{4 \mu }{\pi } \frac{b^{2}+c^{2}}{b^{3} c^{3}}, \end{aligned}$$

which can be expressed alternatively as a function of the cross-sectional area *A* and the aspect ratio $\beta (z) = b(z)/c(z)$, as

33 $$\begin{aligned} \mathcal {R}_0 = \frac{4 \pi \mu (\beta ^{2}+1)}{\beta }\frac{1}{A^2}. \end{aligned}$$

From this expression, we see that the local resistance (per unit length) depends only on the geometry of the local cross section and is independent of axial changes in the geometry. That is, the local resistance is the same as that of a uniform duct of the same cross section.

**Extension to second order.** By extending the lubrication model to higher orders, we anticipate that the solution will include effects of axial variations in cross-sectional geometry, and hence will be in closer agreement with three-dimensional numerical solutions. This has been demonstrated for two-dimensional channels [19]. The second-order dimensional equations are as follows:

34 $$\begin{aligned} 0=\, & {} \frac{\partial {u}_2}{\partial {x}}+\frac{\partial {v}_2}{\partial {y}}+\frac{\partial {w}_2}{\partial {z}}, \end{aligned}$$

35 $$\begin{aligned} 0= & {} -\frac{\partial {p}_2}{\partial {x}} + \mu \left( \frac{\partial ^2}{\partial {x}^2} + \frac{\partial ^2}{\partial {y}^2}\right) {u}_0 , \end{aligned}$$

36 $$\begin{aligned} 0= & {} -\frac{\partial {p}_2}{\partial {y}} + \mu \left( \frac{\partial ^2}{\partial {x}^2} + \frac{\partial ^2}{\partial {y}^2}\right) {v}_0 , \end{aligned}$$

37 $$\begin{aligned} 0= & {} -\frac{\partial {p}_2}{\partial {z}} + \mu \left( \frac{\partial ^2}{\partial {x}^2} + \frac{\partial ^2}{\partial {y}^2}\right) {w}_2+\mu \frac{\partial ^2 {w}_0}{\partial {z}^2}. \end{aligned}$$

The working of these equations yields lengthy expressions, so we relegate the details of the second-order solutions for velocity and pressure to Appendix 2. The resulting expression for pressure gradient is:

38 $$\begin{aligned} \frac{{\partial p_{2} }}{{\partial z}} = & \frac{{\mu Q}}{{3\pi b^{5} c^{5} }}(b^{2} c^{2} \left( {2b^{\prime^2}(6x^{2} - 7c^{2} ) + (6y^{2} - c^{2} )(2c^{\prime^2} - cc^{\prime\prime})} \right) \\ & + \,b^{4} \left( { - 2c^{2} (b^{\prime^2} + 7c^{\prime^2}) + c(c^{2} - 18y^{2} )c^{\prime\prime} + 72y^{2} c^{\prime^2}} \right) \\ & + \,72x^{2} c^{4} b^{\prime^2} + b^{3} c\left( {cb^{\prime\prime}(c^{2} - 6x^{2} ) + 6b^{\prime}c^{\prime}\left( {3(x^{2} + y^{2} ) - c^{2} } \right)} \right) \\ & + \,3bc^{3} \left( {b^{\prime}c^{\prime}\left( {6(x^{2} + y^{2} ) - c^{2} } \right) - 6x^{2} cb^{\prime\prime}} \right) + b^{5} c(cb^{\prime\prime} - 3b^{\prime}c^{\prime})), \\ \end{aligned}$$

where primes denote derivatives with respect to *z*. Here the axial pressure gradient additionally depends on *x* and *y*. Since the pressure distribution over the cross section is difficult to measure, a more relevant quantity is the axial pressure gradient averaged over the cross section,

39 $$\begin{aligned} \left<\frac{\partial p_2}{\partial z}\right>=\frac{1}{\pi b c} \int _A \frac{\partial p_2}{\partial z} \ dA , \end{aligned}$$

which is a function only of *z*. Then we can define the second-order resistance per unit length as $\mathcal {R}_2 \equiv -\left<\partial p_2/\partial z\right>/{Q}$, given by

40 $$\begin{aligned} \mathcal {R}_2= & {} -\frac{\mu }{6 \pi b^4c^4} \big [3 c^4 b'c'+b^3 c \left( 2 b'^2-7 c c''+8 c'^2\right) \nonumber \\{} & {} + b c^3 \left( 8 b'^2-c c''+2 c'^2\right) +b^4 \left( 3 b' c'-c b''\right) \nonumber \\{} & {} + b^2 c^2 \left( 6 b' c'-7 c b''\right) \big ]. \end{aligned}$$

This higher-order resistance depends on the size of the duct and also on the slope and curvature of the walls. It combines linearly with the lower-order resistance, such that the total resistance per unit length is $\mathcal {R}_0 + \alpha ^2 \mathcal {R}_2 + O(\alpha ^4)$. We can express $\mathcal {R}_2$ as a function of area *A* and aspect ratio $\beta$, as we did for $\mathcal {R}_0$, if we further assume that the aspect ratio is fixed at some value $\beta = \beta ^*$, i.e., the cross sections are self-similar along the length of the duct and $d\beta /dz = 0$: the resistance is then

41 $$\begin{aligned} \mathcal {R}_{2, \text {fixed}~\beta } = -\frac{\mu }{ 12 \beta ^2 A^2} \big [\frac{3A'^2}{A}(1+6\beta ^2+\beta ^4)-A''(1+14\beta ^2+\beta ^4)\big ]. \end{aligned}$$

Thus the fractional error of the uniform duct ($0^{\mathrm{th}}$ order) approximation in the total resistance as predicted by the extended lubrication model is

42 $$\begin{aligned} \mathcal {E}=\, & {} \frac{\alpha ^2 \mathcal {R}_2 + O(\alpha ^4)}{\mathcal {R}_0 + \alpha ^2\mathcal {R}_2 + O(\alpha ^4)}\nonumber \\{} & {} \approx (\alpha ^2 \frac{\mathcal {R}_2}{\mathcal {R}_0} + O(\alpha ^4)) (1-\alpha ^2 \frac{\mathcal {R}_2}{\mathcal {R}_0} + O(\alpha ^4)) \nonumber \\{} & {} \approx \alpha ^2 \frac{\mathcal {R}_2}{\mathcal {R}_0} + O(\alpha ^4)\nonumber \\{} & {} \approx \frac{\alpha ^2}{48 \pi \beta (\beta ^2+1)}\left( -\frac{3A'^2}{A}(6\beta ^2+\beta ^4+1)+ A''(\beta ^4+14\beta ^2+1)\right) \nonumber \\{} & {} + O(\alpha ^4) . \end{aligned}$$

If the change in area is relatively small along the length of the duct, i.e., if

$$\begin{aligned} A = A_0(1+\varepsilon g(z)), \quad \varepsilon \ll 1 \end{aligned}$$

then we can show that, to leading order in $\varepsilon$, the second derivative of the area dominates the error:

43 $$\begin{aligned} \mathcal{E} \approx & \frac{{\alpha ^{2} A_{0} }}{{48\pi \beta (\beta ^{2} + 1)}}\left( { - \frac{{3\varepsilon ^{2} g^{{{\prime }2}} }}{{1 + \varepsilon g}}(6\beta ^{2} + \beta ^{4} + 1) + \varepsilon g^{\prime\prime}(\beta ^{4} + 14\beta ^{2} + 1)} \right) \\ \approx & \frac{{\alpha ^{2} }}{{48\pi \beta (\beta ^{2} + 1)}} \\ & \left( { - 3A_{0} \varepsilon ^{2} g^{{{\prime }2}} (1 - \varepsilon g)(6\beta ^{2} + \beta ^{4} + 1) + A_{0} \varepsilon g^{\prime\prime}(\beta ^{4} + 14\beta ^{2} + 1)} \right) \\ \approx & \frac{{\alpha ^{2} (\beta ^{4} + 14\beta ^{2} + 1)}}{{48\pi \beta (\beta ^{2} + 1)}}A^{\prime\prime} + O(\varepsilon ^{2} ). \\ \end{aligned}$$

If we consider this $\beta$-dependent prefactor, we can show that it is a fairly weak function of $\beta$ and its value is within 13.3% of the $\beta = 1$ case for $\beta < 7$. For $\beta = 1$, $\mathcal {E} \approx {\alpha ^2 A''/6\pi }$.

### Approximations using a series of uniform ducts (series unidirectional approach)

Here we investigate the suitability of approximating the hydraulic resistance to flow in an actual perivascular space by modeling it as a series of uniform ducts. Each duct in the series is constructed to have a cross-sectional shape identical to a corresponding section of the PVS, as imaged in 3D. Flow in each is assumed to be unidirectional; we neglect the presence of off-axis (not parallel to the centerline) velocity components. Such off-axis velocity components can arise from axial variations in cross-sectional area or shape, or from curvature of the central axis, and would serve to increase the rate of shear at the wall, thereby increasing the hydraulic resistance compared to that in a unidirectional flow. We refer to this approach as the “series unidirectional” approach.

We compute the velocity profile of unidirectional flow along each duct in the series numerically, by solving Eq. 26 using Matlab’s PDE solver, “solvepde”, as described in [12, 15]. We refer to this solution of the series unidirectional approach as the SUN approach. The mesh size is chosen so that the computed resistance for a circular duct matches the analytically-known value within 0.5%. Instead, for simple cross-sectional shapes like circles, we do the same calculation analytically, referring to this solution as the SUA approach. Both approaches allow us to calculate a resistance per unit length at many locations along the PVS.

**Fig. 2:**
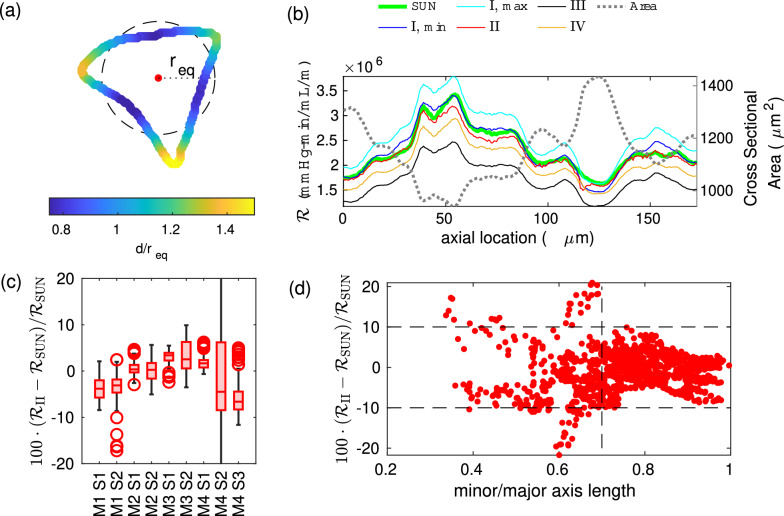
**a** Distance *d* from the center, normalized by $r_{\mathrm eq}$, the radius of a circle with the same area (shown dashed), for an example cross section. **b** Resistance at each cross section, calculated using the series unidirectional approach either by solving Poisson’s equation (“SUN”) or using one of the approximations discussed in the text. **c** Error in approximation $\mathcal {R}_{\mathrm{II}}$ for each segment. (d) Error in $\mathcal {R}_{\mathrm{II}}$ as a function of cross-sectional aspect ratio. If the ratio of the lengths of the minor and major axes exceeds 0.7, the error in $\mathcal {R}_{\mathrm{II}}$ is less than 10%; $\mathcal {R}_{\mathrm{II}}$ is a reasonable estimate when the shape is not too oblong

**Table 1:** Summary of the different solution methods to the series unidirectional approach, where the resistance per unit length is calculated for flow in a series of straight ducts with constant cross section and unidirectional flow

Methods	Description
SUN	Numerically solve Poisson’s equation for resistance
SUA	Analytically solve for the resistance in a circular cross section
I	Solve based on a reference area and resistance, assuming resistance scales with area
II	Adjust $\mathcal {R}_{\mathrm{SUA}}$ using shape factor $\gamma$ based on the distance from the wall to the center
III	Lubrication approximation for an ellipse
IV	Approximation proposed by Bahrami et al.; uses the polar moment of inertia

In addition to solving Poisson’s equation, we also approximate the series unidirectional resistance per unit length using four different solution methods, which we refer to as methods I, II, III, and IV, and which are summarized in Table 1. These methods require considerably less computational effort than solving Poisson’s equation for each cross section (the SUN method). In method I, we predict the series unidirectional resistance per unit length at each normal cross section *i* at $z=z_i$, based on the resistance in a single reference cross section with resistance per unit length $\mathcal {R}_{\mathrm{ref}}$ and area $A_{\mathrm{ref}}$, and the local area $A_i$:

44 $$\begin{aligned} \mathcal {R}_{\mathrm{I}} = \mathcal {R}_{\mathrm{ref}} \left( \frac{A_{\mathrm{ref}}}{A_i} \right) ^2 \end{aligned}$$

The results depend on the reference cross section, so we examine the impact of its selection by choosing two different reference cross sections: the largest and smallest in the PVS segment. The two resulting resistance predictions are denoted $\mathcal {R}_{\mathrm{I,max}}$ and $\mathcal {R}_{\mathrm{I,min}}$, respectively. This approach is based on the idea that the shape of a PVS segment is relatively uniform along its length, so changes in local resistance are due to changes in area alone. We see this illustrated in the lubrication approximation for $\mathcal{R}_0$, Eq. 33, where if the shape factor coefficient is constant, then the resistance depends primarily on $A^{-2}$.

Method II is based on the assumption that radial velocity variation in a cross section of arbitrary shape is similar to that in a circle, such that the shear stress at the wall depends primarily on its distance from the centerline. In this method, we can predict the resistance per unit length of each cross section in the series based on the geometry alone, by multiplying the expression for hydraulic resistance in Hagen-Poiseuille flow in a circular duct by a shape factor $\gamma$ that accounts for the non-circularity of the shape. Thus,

45 $${\mathcal{R}}_{{{\text{II}}}} = \frac{{8\mu }}{{\pi r_{{{\text{eq}}}}^{4} }}\gamma ,{\text{ }}$$

where $r_\mathrm{eq}$ is the radius of a circular cross section of equivalent area and

46 $$\gamma = \frac{1}{N}\sum\limits_{{j = 1}}^{N} {\left( {\frac{{r_{{{\text{eq}}}} }}{{d_{j} }}} \right)^{4} } ,{\text{ }}$$

where the boundary of the cross section has been approximated as a polygon with *N* vertices, each a distance $d_j$ from the center (see Fig. 2a). The center is defined as the location that minimizes $\gamma$ and is typically close to the location of maximum flow velocity. This is appropriate because $d_j$ approximates the radius of a circle for which the wall shear matches that of the realistic cross section at location *j*. (We originally used the geometric centroid of the cross section as the center point but found that when the cross section narrowed in the center, resistance was considerably overestimated.)

Method III predicts the hydraulic resistance of each duct in the series using the lubrication model for an ellipse, as described in Equation 33:

47 $$\begin{aligned} \mathcal {R}_{\mathrm{III}} = \frac{4 \mu }{\pi r_\mathrm{eq}^4} \frac{\beta ^2 +1}{\beta }. \end{aligned}$$

where $\beta$ is now the aspect ratio of an ellipse with the same second moment as the cross section, and *A* is the cross-sectional area.

Method IV is an approximation proposed by Bahrami et al. [21] that considers the shape effect through the second polar moment of inertia $I_p$:

48 $$\begin{aligned} \mathcal {R}_{\mathrm{IV}} = \frac{16 \pi ^2 \mu }{A^4} I_p, \end{aligned}$$

where

49 $$\begin{aligned} I_p = \int _A ((x-x_0)^2 + (y-y_0)^2) \ \mathrm{d}A, \end{aligned}$$

and ($x_0$,$y_0$) are the coordinates for the centroid.

## Results

### Error in the series unidirectional approximation

We calculated the resistance per unit length for PVS segment S2 from mouse M1, and the results are shown in Fig. 1d. We refer to the original geometries as realistic, in contrast to the contrived geometries with circular cross sections but the same area as a function of axial distance. The resistance is calculated by solving numerically or analytically for the resistance in a series of straight ducts with unidirectional flow with cross sections matching those from the 3D geometries, or else calculated from the three-dimensional flow field. Since a circle has the smallest resistance possible for a given cross-sectional area, the resistances for the circular geometries are predictably smaller than those of the realistic geometries. For both the circular and realistic geometries, the series unidirectional approximation of resistance is reasonably close to the resistance from the 3D solution for many cross sections. In Fig. 1e, we show box plots of the error in resistance from the series unidirectional approximation (methods SUN and SUA) relative to the 3D simulations for each geometry. The error is less than 20% in the majority of cases. In Table 2, we report the average resistance per unit length for each of the geometries from the 3D simulations. The average resistance was computed by averaging the resistance from all of the cross sections along the length of the duct. We also report the error in the average resistance between the series unidirectional approximation and the 3D simulations. Resistance is overestimated by the series unidirectional approximation in some cases, and underestimated in others, so the error in the average resistance is smaller than the typical error in a single cross section, which we estimated using the root-mean-square (RMS) value. The series unidirectional approximation underestimates the average resistance by 0.5 to 13% for realistic geometries, and 1.2 to 8.6% for circular geometries.

**Table 2:** Error in resistance per unit length, for all 9 PVS segments, as calculated with the series unidirectional approach, without and with the $\lambda$ correction. Reference resistances $\mathcal {R}_{\mathrm{3D}}$ are not local values, but are averages over the entire segment. RMS errors are enclosed in parentheses; average errors are not

		Realistic		Circular	
segment	$\mathcal {R}_{\mathrm{3D}}$ (mmHg$\cdot$min /mL/m)	($\mathcal {R}_{\mathrm{SUN}}$- $\mathcal {R}_{\mathrm{3D}}$) /$\mathcal {R}_{\mathrm{3D}}$ (%)	($\mathcal {R}_{\mathrm {SUN\cdot \lambda }}$- $\mathcal {R}_{\mathrm{3D}}$) /$\mathcal {R}_{\mathrm{3D}}$ (%)	($\mathcal {R}_{\mathrm{SUA}}$- $\mathcal {R}_{\mathrm{3D}}$) /$\mathcal {R}_{\mathrm{3D}}$ (%)	($\mathcal {R}_{\mathrm {SUA\cdot \lambda }}$- $\mathcal {R}_{\mathrm{3D}}$) /$\mathcal {R}_{\mathrm{3D}}$ (%)
M1 S1	2.8$\times 10^{6}$	− 5.3 (10.4)	− 1.2 (5.5)	− 2.3 (10.4)	0.1 (5.4)
M1 S2	2.4$\times 10^{6}$	− 4.5 (10.2)	− 1.0 (6.2)	− 1.7 (10.8)	− 0.7 (5.8)
M2 S1	8.9$\times 10^{5}$	− 2.8 (4.7)	0.9 (2.5)	− 1.8 (4.7)	− 0.9 (3.4)
M2 S2	8.9$\times 10^{5}$	− 0.5 (6.3)	3.0 (5.1)	− 1.8 (8.4)	− 1.7 (5.4)
M3 S1	2.1$\times 10^{6}$	− 7.1 (12.4)	− 2.7 (9.3)	− 8.6 (16.4)	− 5.2 (14.4)
M3 S2	3.5$\times 10^{6}$	− 9.5 (15.5)	− 4.5 (10.8)	− 6.0 (14.4)	− 3.3 (11.9)
M4 S1	1.7$\times 10^{6}$	− 4.6 (9.8)	− 0.8 (5.4)	− 1.4 (8.3)	− 0.1 (4.7)
M4 S2	9$\times 10^{6}$	− 12.6 (31.1)	− 8.2 (30.3)	− 5.4 (24.6)	− 0.6 (16.1)
M4 S3	5.5$\times 10^{5}$	− 3.5 (11.5)	− 0.2 (6.4)	− 1.2 (16.0)	0.7 (11.5)

### Alternative solutions to the series unidirectional approximation

We show the results for methods I, II, III, and IV in Fig. 2b and Table 3. All four approaches require considerably less computational power than numerically solving Poisson’s equation (shown in green in Fig. 2b for comparison), and are reasonable approximations.

Depending on the reference cross section, method I produces errors in average resistance between − 8 and 22%, with RMS errors between 2 and 31%. The error is calculated with respect to the SUN approach and does not include the additional error inherent in the series unidirectional approximation in the first place.

Method II performs the best of all the methods, with errors in average resistance between − 7 and 5%, and RMS errors between 2 and 13%. In Fig. 2c we show a box plot of the error in $\mathcal {R}_{\mathrm{II}}$ (with respect to $\mathcal {R}_{\mathrm{SUN}}$, the resistance per unit length calculated using the SUN approach), which shows that for most cross sections, the errors are less than 10%. This approach breaks down if the cross section is very oblong, such that the peak velocity in the cross section does not occur at a single central point, but along a ridge. To quantify this, we plot error as a function of the minor to major axis ratio of an ellipse with the same second moment as each cross section ($1/\beta$) in Fig. 2d, and find that when this ratio is greater than 0.7, the error is always less than 10%.

Method III underestimates the (average) resistance by between 8 and 25%. This makes sense, since it is derived for an elliptical duct, which typically would a have lower resistance than many of these “realistic” cross sections, some of which are concave in spots, and generally not as smooth as an ellipse, as described by Racivic et al. [12]. The average value of $\beta$ for each geometry ranges between 1.1 and 2, so an ellipse with the same $\beta$ is similar to a circle, which has the minimum resistance for a given area. Accordingly, the $(\beta ^2+1)/\beta$ term that is the ellipse correction factor is typically close to one.

Method IV underestimates the average resistance by between 5 and 13%, depending on the geometry.

**Table 3:** Error in resistance per unit length, for all 9 PVS segments, as calculated with approaches I, II, III, and IV. RMS errors are enclosed in parentheses; average errors are not

segment	( $\mathcal {R}_{\mathrm{I,max}}$– $\mathcal {R}_{\mathrm{SUN}}$) /$\mathcal {R}_{\mathrm{SUN}}$(%)	( $\mathcal {R}_{\mathrm{I,min}}$– $\mathcal {R}_{\mathrm{SUN}}$) /$\mathcal {R}_{\mathrm{SUN}}$(%)	( $\mathcal {R}_{\mathrm{II}}$−$\mathcal {R}_{\mathrm{SUN}}$) /$\mathcal {R}_{\mathrm{SUN}}$(%)	( $\mathcal {R}_{\mathrm{III}}$− $\mathcal {R}_{\mathrm{SUN}}$) /$\mathcal {R}_{\mathrm{SUN}}$(%)	( $\mathcal {R}_{\mathrm{IV}}$- $\mathcal {R}_{\mathrm{SUN}}$) /$\mathcal {R}_{\mathrm{SUN}}$(%)
M1 S1	1.8 (3.2)	3.2 (4.2)	− 3.9 (4.8)	− 25.3 (25.8)	− 12.5 (12.8)
M1 S2	1.0 (4.4)	12.4 (13.7)	− 3.4 (4.5)	− 25.1 (25.8)	− 12.3 (12.7)
M2 S1	− 3.0 (3.6)	− 1.8 (2.7)	0.9 (2.1)	− 16.1 (16.5)	− 9.3 (9.5)
M2 S2	4.0 (5.2)	− 0.6 (2.9)	1.2 (3.1)	− 17.3 (20.0)	− 9.6 (11.1)
M3 S1	− 1.6 (2.7)	9.8 (12.0)	3.3 (3.9)	− 7.8 (8.5)	− 5.2 (5.6)
M3 S2	− 8.3 (9.6)	22.2 (31.1)	4.6 (7.2)	− 10.7 (10.9)	− 6.9 (7.1)
M4 S1	0.6 (3.2)	− 0.1 (3.1)	1.9 (2.5)	− 18.8 (19.2)	− 10.2 (10.4)
M4 S2	− 8.1 (18.3)	6.2 (19.9)	1.8 (13.3)	− 28.2 (31.7)	− 11.1 (12.6)
M4 S3	3.9 (6.1)	5.2 (7.0)	− 6.7 (8.4)	− 22.2 (23.9)	− 11.0 (11.6)

### Test case: a duct with circular cross section with sinusoidally varying radius

**Fig. 3:**
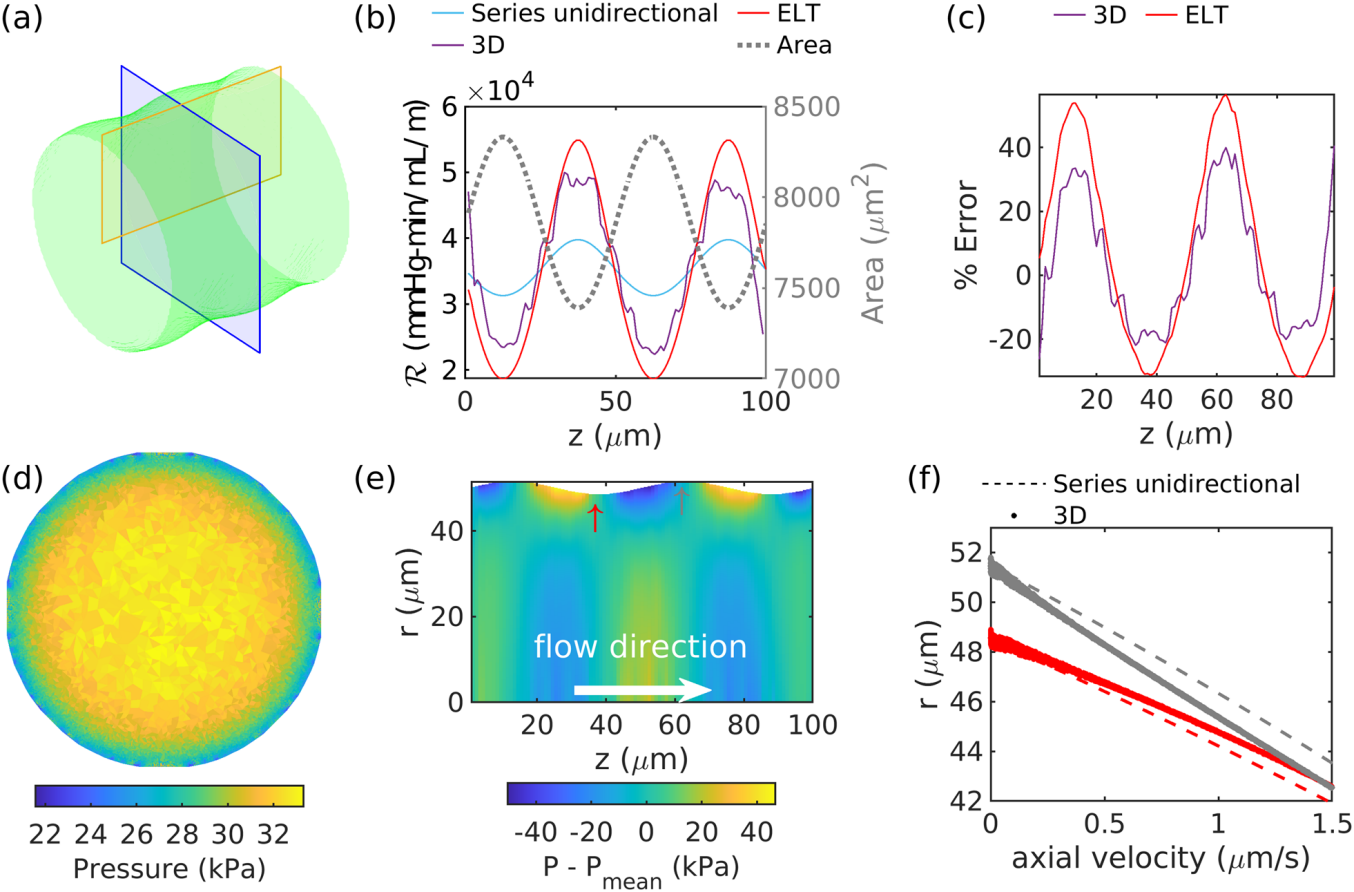
**a** Duct with circular cross sections and sinusoidally varying radius. **b** Resistance per unit length as calculated from three−dimensional simulations (“3D”), from the series unidirectional approach, and from extended lubrication theory (“ELT”), along with cross-sectional area. **c** Error in resistance per unit length predicted by the series unidirectional approximation, compared to 3D simulations and extended lubrication theory. **d** Pressure at the cross section marked with a blue box in (a). **e** Radial pressure fluctuations in the longitudinal plane marked with a yellow box in (a). **f** Axial velocity profiles at the widest (gray) and narrowest (red) locations in the duct, marked by arrows in **e**. The error in the series unidirectional approach can be predicted with extended lubrication theory and results from the radial pressure variations induced by the axial variation in area

In order to gain insight into the aspects of the 3D geometry that are not captured in the series unidirectional approximation, we studied the simpler case of a duct having a circular cross section with radius varying sinusoidally along its axis according to

50 $$\begin{aligned} r=50+1.5\, \mathrm{sin}(2\pi z/50) , \end{aligned}$$

where *r* is the radius (measured in µm, as is *z*). The resulting shape is shown in Fig. 3a. The resistance per unit length as calculated using the series unidirectional approximation (which can be solved analytically for a circular cross section) and the 3D solution are shown in Fig. 3b, and the fractional error between these two results is shown in Fig. 3c. The series unidirectional calculation overestimates the resistance in wide regions and underestimates it in narrow ones.

We can predict the difference in resistance between a duct with axially varying area and one with constant area using extended lubrication theory. In Figs 3b and c, we plot the resistance and error predicted using lubrication theory, and it agrees well with the 3D solution. The good agreement shows that the $2^{\mathrm{nd}}$-order extension to the lubrication approximation captures the majority of the difference in resistance between the series unidirectional approach (derived from the $0^{\mathrm{th}}$-order lubrication approximation) and the full 3D solution. Importantly, from the extended lubrication theory, we learn that the error in the series unidirectional approach scales with the second derivative of the area. Some of the difference between the $2^{\mathrm{nd}}$-order extended lubrication approximation and 3D resistances can be attributed to the discretization of the domain boundaries, the presence of higher order effects not captured in the $0^{\mathrm{th}}$-order lubrication approximation, and the difference in how the $\mathcal {R}$ is calculated between the 3D simulations and ELT (discussed in Appendix 3), though the latter should account for a difference on the order of just 3% for this case.

Here we explain why the resistance differs between the 3D and series unidirectional approaches. The resistance is a measure of the pressure drop required to move fluid through a duct at a certain rate, and it is calculated by measuring the decrease in pressure, or pressure gradient, in the axial direction. The pressure gradient is a reflection of the shear rate at the wall, which is dictated by the velocity gradient. Axial area variations change the velocity gradient, shear rate at the wall, pressure gradient, and resistance, relative to a uniform duct. The velocity, and thus velocity gradient, are dictated by the pressure. In Fig. 3d, we show the pressure from the 3D solution in one cross section of the duct shown in panel (a); there is a clear radial pressure variation. In Fig. 3e, we show the radial pressure fluctuation $P - P_{\mathrm{mean}}(z)$ (where $P_{\mathrm{mean}}(z)$ is the radially-averaged pressure), which excludes the mean axial pressure gradient that would otherwise dominate. The radial pressure fluctuation modifies the axial velocity at the wall. For example, where the wall bulges inward at *z* = 37 µm, relative to a scenario with a duct with the same size and constant cross section, there is a higher pressure region upstream, and a lower pressure region downstream. This negative pressure gradient steepens the axial velocity gradient, as shown in Fig. 3f, resulting in larger shear rates and larger pressure gradients, relative to a scenario with a duct with the same size and constant cross section. Thus, the series unidirectional approach underestimates the resistance at constrictions. Where the wall bulges outward, the opposite is true: pressure is lower upstream and higher downstream, flattening the axial velocity gradient and reducing shear (as shown in Fig. 3f). Thus, the series unidirectional approach overestimates the resistance where the cross-sectional area is locally maximum.

### The error in the series unidirectional approximation correlates with $d^2A/dz^2$

**Fig. 4:**
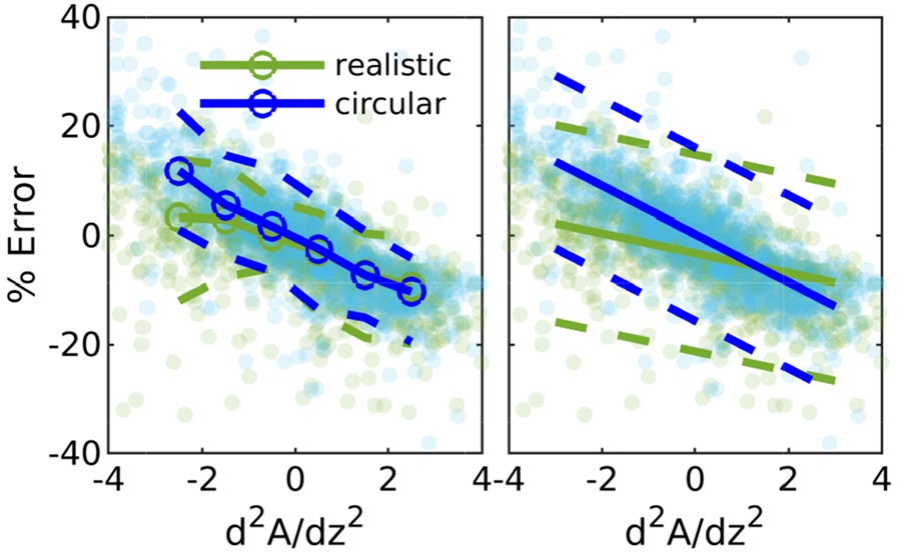
Error in resistance between the series unidirectional approximation and the three-dimensional solution for all cross sections, as a function of the second derivative of the cross sectional area with respect with the axial direction. Pale markers indicate values for individual cross sections. In the left panel, curves indicate the $5^{\mathrm{th}}$ (dashed), $50^{\mathrm{th}}$ (solid), and $95^{\mathrm{th}}$ (dashed) percentiles. In the right panel, solid lines show linear fits, and dashed lines indicate 95% confidence bounds. The error correlates strongly with the second derivative of the area for both the realistic and circular cross sections, though the correlation is slightly stronger for the circular cross sections. Across all normal cross sections, for the realistic cross sections the Pearson correlation coefficient $\rho$ = − 0.49 and *p* value < 0.0001, and for the circular cross sections $\rho$ = − 0.80, $p<0.0001$

From the extended (second order) lubrication model, we know that for a duct with circular cross sections, the error from the series unidirectional approach approximately scales with the second derivative of the cross-sectional area with respect to axial distance. In Fig. 4 we show the error as a function of $d^2A/dz^2$. As expected, there is a clear correlation for the ducts with circular cross sections (*p* value < 0.0001). For the realistic geometries, the variation in area is not radially uniform, and the change in area does not fully capture how the geometry changes; despite this, however, there is still a strong correlation ($p<0.0001$).

In order to quantify the uncertainty associated with this correlation, we also show conditional statistics for scattered data, with the circles showing the median error, binned according to $d^2A/dz^2$. The dashed lines indicate the 5th and 95th percentiles.

We fit a first-order polynomial to the data (shown in the right panel in Fig. 4), and the equations of the fit lines for realistic and circular shapes are $\mathrm{err}_{\mathrm{real}} = -1.8\cdot d^2A/dz^2 -3.2$ and $\mathrm{err}_{\mathrm{circ}} = -4.4\cdot d^2A/dz^2 + 0.22$, respectively. The slope of the fit line for the geometries with circular cross sections is -4.4, which is close to the slope of $100/(6\pi )$, or -5.3, predicted from extended lubrication theory.

We used the equation of the fit lines to find a correction factor $\lambda$ for the series unidirectional approach that accounts for the three-dimensional nature of the flow, and we plotted the resulting predicted resistances, $\mathcal {R}_{\mathrm {SUA\cdot \lambda }}$ and $\mathcal {R}_{\mathrm {SUN\cdot \lambda }}$, in Fig. 1d, with corresponding errors in Fig. 1e. We report the average and RMS error in Table 2. This correction factor reduces the error significantly, by more than half for most of the geometries.

## Discussion

We gained insight into those aspects of the 3D configuration that result in errors in the series unidirectional approach by studying a circular duct with sinusoidally varying radius. We found that the error (between the series unidirectional approximation and the full 3D solution) is largest at locations where the second derivative of the radius with respect to the axial distance has maximum magnitude, or in other words, where the slope of the channel wall (relative to the central axis) is changing most rapidly. We used extended lubrication theory to define a correction factor for the series unidirectional approximation for a circular duct, and it agrees well with the full 3D simulations.

There is some error introduced by the discretization of the geometry, which is determined by the spatial resolution of the microscope images. For both the 2D and 3D numerical simulations, we reduced the internal mesh size until the errors were quite small, but the external domain boundaries remained the same. The non-smooth variation of the resistance in the sinusoidal geometry is evidence of this discretization error, which is accentuated when taking the numerical derivative of the pressure.

None of our simulations account for any increase in hydraulic resistance due to curvature of the centerline of a PVS. However, for these slow viscous flows this effect is generally very small and negligible. For duct flows in general, the contribution of off-axis flow components arising from centerline curvature is usually measured by the Dean number *De*, a dimensionless group relating inertial and centripetal forces to viscous forces in a flow: $De \equiv Re \sqrt{r_\mathrm{e}/r_\mathrm{c}}$ where *Re* is the Reynolds number, $r_\mathrm{e} \equiv \sqrt{A/\pi }$ is the radius of a circle with the same area, and $r_\mathrm{c}$ is the radius of curvature of the vessel centerline. Flows in pial PVSs have Reynolds numbers on the order of $10^{-3}$ [22], and these PVSs have cross-sectional areas of about 100 µm$^2$, or $r_\mathrm{e}$ $\approx$ 6 µm. In the PVS geometries used in this work, the average value of $r_\mathrm{c}$ is about 400 µm and is rarely less than 100 µm. Therefore, pial PVSs are expected to have a maximum Dean number of $De \approx 2.5 \times 10^{-4}$, and hence the contribution of centerline curvature to the resistance is clearly negligible [23].

We constructed circular ducts with varying cross-sectional area that matched the variation in area in the realistic geometries in order to determine how much the variation in shape along the duct affects the accuracy of the series unidirectional approach. One could imagine a duct that maintained nearly constant cross-sectional area, but where the cross-sectional shape changed from a circle to a square or a triangle. Depending on the rate of change, this change in shape alone could introduce significant off-axis velocity components and errors in the series unidirectional approach. In the realistic ducts, both the cross-sectional area and the shape vary in the axial direction, but in the circular ducts only the area varies, so the difference in error between the two cases can be attributed to the change in shape. As evident in Fig. 1e and Table 2, the average and RMS error are comparable in the two types of ducts, and either one can be larger.

In the circular ducts, the non-axial velocity components and the radial variation in pressure are axisymmetric, but that does not necessarily minimize the error in the series unidirectional approach. One could imagine a situation where the change in area and shape occur only within a very narrow azimuthal range, such as at a sudden and narrow protrusion on one side of the duct. In this case, the error in the series unidirectional approach for the realistic geometry might be less than that in the circular geometry, because the effects causing the error are concentrated near a small portion of the boundary. For realistic PVSs, variations in shape and area do not necessarily induce significantly more error in the series unidirectional approach than does the variation in area alone.

For most practical applications, including modeling flow in PVSs, the total resistance *R*, rather than the local resistance per unit length $\mathcal {R}$, is the quantity of interest. For 3D simulations, *R* can be calculated directly, but 3D simulations are computationally expensive and impractical for simulating flow through large systems of ducts like the glymphatic system. For the series unidirectional approach, *R* can be obtained by averaging $\mathcal {R}$ and multiplying by the length of the duct: the error is then the average resistance error given in Tables 2 and 3. The error in the series unidirectional approach for any single cross section is likely to be larger than the average error, as indicated by the RMS error, since the series unidirectional approach sometimes overestimates and sometimes underestimates the resistance.

On average, the series unidirectional approach always underestimates the resistance in a realistic PVS because the non-axial velocity components generated by changes in duct area and cross section increase the shear rate at the wall. This is reflected in the nonzero constant term in the linear fit describing the error as a function of the second derivative of the area. The constant is much larger for realistic ducts than that for circular ducts, suggesting that the change in shape gives rise to the nonzero average error. The nonzero average error is present in the extended lubrication theory through the term containing the first derivative; this term is small compared to the second derivative, but generally nonzero when the second derivative is zero.

The realistic ducts analyzed here were all derived from murine pial PVSs in the vicinity of the middle cerebral artery, and thus the error magnitudes and correction factors calculated here are most appropriately applied to estimate the error in neglecting off-axis flow velocities in PVSs in similar locations, since it is unclear how the shape of PVSs in other locations (and species) may differ. Further, the analysis here is only applicable to PVSs that are predominantly open, as pial PVSs have been shown to be [13], rather than filled with connective tissue and porous, as penetrating PVSs are likely to be [14]. We describe in detail how porous PVSs can be treated in Appendix 1. However, one point can be extended to open PVSs in other locations and to any duct with low Reynolds, Womersley, and Dean numbers and where the duct is much longer than it is wide: regardless of shape, the error in neglecting off-axis velocity components scales with the second derivative of the area with respect to axial distance, as we show with the lubrication approximation. This principle can be used to estimate the error that arises from neglecting off-axis velocity components regardless of the shape of the duct, as long as the shape of the duct remains relatively constant. The exact correction factor may differ for differently shaped PVSs, but the existence of a scaling will translate. At present, it is unclear how much variation in shape exists in PVSs at other locations and across species, but we suppose that the error will in general scale with the second derivative of the area.

Additionally, we show that approach II typically has errors less than 10% as long at the ratio of the minor-to-major axis is less than 0.7. We expect this would be true for a wide variety of mostly convex shapes so that approach II could likely be used to estimate the resistance in many types of PVSs that have a bi-lobed shape, and we suppose this would be true for pial PVSs in other regions and species. Approach II may not work as well for estimating resistance in penetrating PVSs.

## Conclusions

Assuming a uniform duct and neglecting off-axis velocity components underestimates the average resistance by as much as 15%, depending on the configuration. We gained insight into the aspects of the geometry that cause errors in the series unidirectional approach by studying a circular duct with sinusoidally varying radius, and we showed, using extended lubrication theory, that the error in a circular duct can be predicted based on the second derivative of the area with respect to the axial distance. We showed further that the error in approximating the resistance in realistic, non-circular ducts using the series unidirectional approach correlates strongly with the second derivative of the area. We can predict this error, with a 95% confidence interval, based solely on this second derivative. We suggested a specific correction factor for the series unidirectional approach based on the second derivative that significantly reduces the error.

We approximated the series unidirectional resistance in four different ways that use considerably less computation power and find that they each predict the resistance reasonably well (errors comparable to those arising from neglecting off-axis velocity components), with little computational cost. The first approach uses the resistance in a single cross section and the cross-sectional area along the length of the duct. The second approach yields a correction factor for ducts of arbitrary cross-sectional area. The third approach is based on the solution for an elliptical duct, with a correction factor that is a function of the major-to-minor axis ratio. The fourth approach uses a correction factor based on the polar moment of inertia of the cross section. Of these four approaches, the second results in the smallest errors, on average.

Based on these results, we make the following recommendations for estimating the hydraulic resistance in an actual PVSs, given its full 3D configuration, or a single cross section. If the 3D configuration is known and resistance is to be predicted without solving the full 3D Navier-Stokes equations, the series unidirectional approach (solve the 2D Poisson’s equation numerically for each cross section, the SUN solution) is a useful method, resulting in average errors on the order of 0 to 20%, depending on the geometry. The error correlates with the second derivative of the cross sectional area, and generally $| d^2A/dz^2 |<$ 2 results in less than 20% error. If the axial area variation is known, the series unidirectional approximation can be improved with the $\lambda$ correction. In order to use less computational power than is required for solving Poisson’s equation numerically, approach II is a useful method, resulting in additional errors $< \pm$ 10%, as long as the minor-to-major axis ratio of the ellipse with the same second moment as the cross sectional shape is less than 0.7.

## Data Availability

The datasets analyzed are described by Boster et al. [15] and Raicevic et al. [12] and are available at https://zenodo.org/record/7723381#.ZAzSZjpKhD8 and https://identifiers.org/DANDI:000491.

## Appendix 1: Flow and hydraulic resistance in porous perivascular spaces

Here we consider PVSs that contain enough connecting tissue that they can be treated as a porous medium, with CSF flow governed by the Darcy equation. Little is currently known about the detailed configurations of these PVSs, so here we just present a method of calculating the hydraulic resistance in anticipation of better data becoming available with improved in vivo imaging techniques. To simplify the analysis, we assume that the size and shape of the cross section vary slowly along the PVS, in which case the non-axial components of velocity make a negligible contribution to the resistance, the axial flow velocity is essentially uniform over the cross section, and the hydraulic resistance per unit length depends only on the area of the cross section.

Consider a PVS in the form of an annular tube whose cross section varies along the tube. Let *z* be an axial curvilinear coordinate running along the tube, and let *A*(*z*) denote the varying internal cross-sectional area of the tube. In this case, the Darcy law for steady flow along the tube has the differential form

51 $$\begin{aligned} Q = - \frac{\kappa }{\mu } \left( \frac{\partial p}{\partial z}\right) A(z) , \end{aligned}$$

where *Q* is the constant volume flow rate, $\kappa$ is the permeability, $\mu$ is the dynamic viscosity, and *p*(*z*) is the pressure, which varies long the tube. Note that the pressure gradient $\partial p / \partial z$ (and hence the hydraulic resistance per unit length) varies inversely with the cross-sectional area *A*(*z*). Now consider a finite section of this PVS of length *L*, running from $z=0$ to $z=L$. The total pressure drop $\Delta p$ along this section is given by

52 $$\begin{aligned} \Delta p = -[p(L) - p(0)] = - \int _0^L \left( \frac{\partial p}{\partial z}\right) dz = \frac{\mu Q}{\kappa } \int _0^L \frac{dz}{A(z)}. \end{aligned}$$

If we define an *effective cross-sectional area* $A_\mathrm{eff}$ by the relation

53 $$\begin{aligned} \frac{1}{A_\mathrm{eff}} \equiv \frac{1}{L}\int _0^L \frac{dz}{A(z)} \ , \end{aligned}$$

then the total pressure drop $\Delta p$ is given by

54 $$\begin{aligned} \Delta p = \frac{\mu Q}{\kappa } \frac{L}{A_\mathrm{eff}} \ , \end{aligned}$$

which is the same as the pressure drop along a uniform tube of constant cross-sectional area $A_\mathrm{eff}$. For an actual PVS segment, if we have experimental data from which we can construct an approximate relation *A*(*z*) for the varying cross-sectional area, then we can calculate $A_\mathrm{eff}$ by numerical integration, which will then give us the pressure drop $\Delta p$ and the total hydraulic resistance *R* of the segment,

55 $$\begin{aligned} R \equiv \Delta p /Q = \frac{\mu }{\kappa } \frac{L}{A_\mathrm{eff}} . \end{aligned}$$

Note that the total hydraulic resistance of the PVS segment depends only on its geometrical configuration (its length *L* and effective cross-sectional area $A_\mathrm{eff}$), its permeability $\kappa$, and the viscosity of the flowing CSF. Note also that in the definition of the effective cross-sectional area, the smallest values of *A*(*z*) (the narrowest parts of the PVS segment, where the flow is most constricted) contribute the most in determining $A_\mathrm{eff}$ and hence the hydraulic resistance *R*.

If the permeability $\kappa$ varies significantly along the PVS segment, we can account for this in estimating the hydraulic resistance. Supposing that the permeability varies as $\kappa (z)$, equation (52) can be replaced by the relation

56 $$\begin{aligned} \Delta p = Q \mu \int _0^L \frac{1}{\kappa (z) A(z)} dz , \end{aligned}$$

which can be evaluated for experimental data by numerical integration.

As a simple model, consider a tube of length *L* with a circular annulus cross section, having a uniform inner radius $r_0$ (representing the surface of the blood vessel) and an outer radius that varies uniformly from $r_1$ at the entrance ($z=0$) to $r_2$ at the exit ($z=L$), i.e., $r(z) = r_1 - \alpha z$, where $\alpha = (r_1 - r_2)/L$. In this case the effective cross-sectional area is

57 $$\begin{aligned} A_\mathrm{eff} = \left[ \int _0^L \frac{dz}{\pi (r_0 - \alpha z)^2 - r_0^2} \right] ^{-1} = \frac{2 \pi r_0 (r_1 -r_2)}{\ln \left[ \left( \frac{r_1 - r_0}{r_1 + r_0}\right) \left( \frac{r_2 + r_0}{r_2 - r_0}\right) \right] } . \end{aligned}$$

Note that this expression holds for both $r_1 > r_2$ (narrowing tube) and $r_1 < r_2$ (expanding tube), corresponding to the reversibility of the direction of Darcy flow. It can be shown that this expression for $A_\mathrm{eff}$ has the limiting form $A_\mathrm{eff} = \pi (r_1^2 - r_0^2)$ when $r_2 = r_1$, i.e, the effective cross-sectional area is equal to the actual cross-sectional area in the case of a uniform annular tube.

To illustrate simply how the narrowest parts of a tube contribute most to its hydraulic resistance, suppose the inner tube of the annulus is absent, i.e. $r_0 = 0$, in which case the effective cross-sectional area is given directly by

58 $$\begin{aligned} A_\mathrm{eff} = \left[ \int _0^L \frac{dz}{\pi (r_1 - \alpha z)^2} \right] ^{-1} = \pi r_1 r_2 , \end{aligned}$$

and it can be shown that the expression (57) for $A_\mathrm{eff}$ of the annular tube has this limiting form for $r_0 \rightarrow 0$. Note that this effective area is the same as that of a uniform tube of radius equal to the geometric mean $\sqrt{r_1 r_2}$ of $r_1$ and $r_2$. The geometric mean radius $\sqrt{r_1 r_2}$ is always less than or equal to the arithmetic mean radius $(r_1 + r_2)/2$, which in turn is always less than or equal to the quadratic mean radius $\sqrt{(r_1^2 + r_2^2)/2}$, i.e.,

59 $$\begin{aligned} \sqrt{r_1 r_2} \le (r_1 + r_2)/2 \le \sqrt{(r_1^2 + r_2^2)/2} \ . \end{aligned}$$

Thus, the effective cross-sectional area $A_\mathrm{eff} =\pi r_1 r_2$ of the tapering tube is always less than the cross-sectional area $A_\mathrm{m} = \pi [(r_1 + r_2)/2]^2$ of a uniform tube with the arithmetic mean radius $(r_1 + r_2)/2$, which in turn is less than the cross-sectional area $A_\mathrm{qm} = \pi (r_1^2 + r_2^2)/2$ of a uniform tube with the quadratic mean radius $\sqrt{(r_1^2 + r_2^2)/2}$. (Note that $A_\mathrm{qm}$ is the average of the areas at each end of the segment.) Hence, $A_\mathrm{eff} \le A_\mathrm{m} \le A_\mathrm{qm}$, and the hydraulic resistances are correspondingly ordered $R_\mathrm{eff} \ge R_\mathrm{m} \ge R_\mathrm{qm}$. (The equal signs correspond to the case $r_1 = r_2$.) This simple example shows the dominating effect of the narrowest sections of a porous PVS in determining its hydraulic resistance.

Returning to the case of the annular tube, the hydraulic resistance is given by

60 $$\begin{aligned} R_\mathrm{eff} = \frac{\mu }{\kappa } \frac{L}{A_\mathrm{eff}} = \frac{\mu }{\kappa } \frac{L}{2 \pi r_0 (r_1 - r_2)} \ln \left[ \left( \frac{r_1 - r_0}{r_1 + r_0}\right) \left( \frac{r_2 + r_0}{r_2 - r_0}\right) \right] . \end{aligned}$$

Now consider an annular tube consisting of a continuous sequence of segments of uniformly varying outer radius, for which the total hydraulic resistance will be the sum of the resistances of the individual segments (analogous to electrical resistors in series). Suppose the tube starts with outer radius $r_1$, and consists of *N* segments with lengths $L_n$, entry radii $r_{n}$, and exit radii $r_{n+1}$, $n = 1,2,3, ..., N$. The hydraulic resistance of the $n^{\mathrm{th}}$ segment is

61 $$\begin{aligned} R_n = \frac{\mu }{\kappa } \frac{L_n}{2 \pi r_0(r_{n+1} - r_{n}}) \ln \left[ \left( \frac{r_n - r_0}{r_n + r_0}\right) \left( \frac{r_{n+1} + r_0}{r_{n+1} - r_0}\right) \right] , \end{aligned}$$

and the total hydraulic resistance of the tube is $R = \sum _1^N R_n$. This model can be used to estimate the hydraulic resistance of a real porous PVS if we approximate its configuration as a connected series of circular annular segments of uniformly varying outer radius. The PVS may not have a circular outer boundary, but we can choose outer radii of the segments so that the areas of the ends of each segment match the actual local cross-sectional area of the PVS. This model can be easily extended to a case in which the porosity varies along the PVS, by allowing for different values $\kappa _n$ in equation (61).

## Appendix 2: Velocity and pressure in the extended lubrication model

The derivation that follows can be deduced from the solution for an elliptical duct [18] and the presentation of the general extended lubrication theory for a 2D channel [19].

To obtain the transverse velocities $u_0$, $v_0$, we combine the $2^{\mathrm{nd}}$ order *x* and *y* momentum equations (Eqs. 35 and 36) to eliminate $p_2$:

62 $$\begin{aligned} \frac{\partial }{\partial y}\left( \left( \frac{\partial ^{2}}{\partial x^{2}}+\frac{\partial ^{2}}{\partial y^{2}}\right) u_{0}\right) -\frac{\partial }{\partial x}\left( \left( \frac{\partial ^{2}}{\partial x^{2}}+\frac{\partial ^{2}}{\partial y^{2}}\right) v_{0}\right) =0. \end{aligned}$$

For this to be satisfied, $\left( \frac{\partial ^{2}}{\partial x^{2}}+\frac{\partial ^{2}}{\partial y^{2}}\right) u_{0}$ must be a function only of *x* (or a constant), and similarly, $\left( \frac{\partial ^{2}}{\partial x^{2}}+\frac{\partial ^{2}}{\partial y^{2}}\right) v_{0}$ must be a function only of *y* (or a constant). Moreover, $u_0$ and $v_0$ likely have a similar form as the axial velocity $w_0$ and must also satisfy no-slip on the boundary. These conditions are satisfied by

$$\begin{aligned} u_{0}=k_1 x \left( -1+\left( \frac{x}{b}\right) ^{2}+\left( \frac{y}{c}\right) ^{2}\right) , \quad v_{0}=k_2 y\left( -1+\left( \frac{x}{b}\right) ^{2} + \left( \frac{y}{c}\right) ^{2}\right) , \end{aligned}$$

and by enforcing continuity we find

63 $$\begin{aligned} u_{0}= & {} -\frac{2Q}{\pi } \frac{b^{\prime }}{b^{2} c} x\left( \left( \frac{x}{b}\right) ^{2}+\left( \frac{y}{c}\right) ^{2}-1\right) , \end{aligned}$$

64 $$\begin{aligned} v_{0}= & {} -\frac{2 Q}{\pi } \frac{c^{\prime }}{b c^{2}} y\left( \left( \frac{x}{b}\right) ^{2}+\left( \frac{y}{c}\right) ^{2}-1\right) . \end{aligned}$$

Next we can solve for the second-order pressure $p_2$ by integrating the *x* and *y* momentum equations:

$$\begin{aligned} p_{2} =C_{1}(z) \mu - \frac{2 Q \mu }{\pi b c}\left( \frac{c^{\prime } y^{2}}{c}\left( \frac{3}{c^{2}}+\frac{1}{b^{2}}\right) +\frac{b^{\prime } x^{2}}{b}\left( \frac{3}{b^{2}}+\frac{1}{c^{2}}\right) \right) , \end{aligned}$$

where $C_1(z)$ is a function to be determined later. We can then obtain the second-order axial velocity $w_2$ from the *z*-momentum equation

$$\begin{aligned} \mu \left( \frac{\partial ^2}{\partial {x}^2} + \frac{\partial ^2}{\partial {y}^2}\right) {w}_2 = \frac{\partial {p}_2}{\partial {z}} - \mu \frac{\partial ^2 {w}_0}{\partial {z}^2}. \end{aligned}$$

By inspection, the right-hand side of this equation is a function of $x^2$ and $y^2$, so we can guess that $w_2$ has the general polynomial form

$$\begin{aligned} w_{2}= \left( \left( \frac{x}{b}\right) ^{2}+\left( \frac{y}{b}\right) ^{2}-1\right) \left( D_{1}+D_{1} x^{2}+D_{2} x^{4}+D_{3} y^{2}+D_{4} y^{4}+D_{5} x^{2} y^{2}\right) . \end{aligned}$$

Substituting this expression in the momentum equation and collecting like terms, we can solve for the constants $D_n$ to yield

$$\begin{aligned} w_2 = \frac{K}{\pi bc}\left( 1-\frac{x^2}{b^2}-\frac{y^2}{c^2}\right) , \end{aligned}$$

where

$$\begin{aligned} \frac{K}{Q} = & - \frac{{4b^{5} cb^{\prime } c^{\prime } + 8b^{3} c^{3} b^{\prime } c^{\prime } + 4bc^{5} b^{\prime } c^{\prime } + 20b^{2} c^{4} b^{{\prime 2}} + 4c^{6} b^{{\prime 2}} + 4b^{6} c^{{\prime 2}} + 20b^{4} c^{2} c^{{\prime 2}} }}{{2\left( {7b^{4} c^{2} + 7b^{2} c^{4} + b^{6} + c^{6} } \right)}} - \frac{{b^{5} 6\pi c^{5} + b^{3} \pi c^{7} + \pi b^{7} c^{3} }}{{2Q\left( {7b^{4} c^{2} + 7b^{2} c^{4} + b^{6} + c^{6} } \right)}}C_1^{\prime } + \\ & \frac{{ - 8b^{2} c^{3} b^{\prime } c^{\prime } - 3c^{5} b^{\prime } c^{\prime } - b^{4} cb^{\prime } c^{\prime } + bc^{4} \left( {2b^{{\prime 2}} + cc^{{\prime \prime }} - 2c^{{\prime 2}} } \right) + b^{5} \left( {cc^{{\prime \prime }} - 4c^{{\prime 2}} } \right) + 6b^{3} c^{2} \left( {cc^{{\prime \prime }} - 4c^{{\prime 2}} } \right)}}{{bc^{2} \left( {6b^{2} c^{2} + b^{4} + c^{4} } \right)}}y^{2} \\ & + \frac{{2b^{4} c\left( {c^{{\prime 2}} - b^{{\prime 2}} } \right) - 24b^{2} c^{3} b^{{\prime 2}} - 4c^{5} b^{{\prime 2}} + b^{5} \left( {cb^{{\prime \prime }} - 3b^{\prime } c^{\prime } } \right) + 2b^{3} c^{2} \left( {3cb^{{\prime \prime }} - 4b^{\prime } c^{\prime } } \right) + bc^{4} \left( {cb^{{\prime \prime }} - b^{\prime } c^{\prime } } \right)}}{{b^{2} c\left( {6b^{2} c^{2} + b^{4} + c^{4} } \right)}}x^{2} . \\ \end{aligned}$$

Finally, we obtain $C_1'(z)$ by applying the integral constraint

65 $$\begin{aligned} 0=4 \int _{0}^{c} \int _{0}^{b \sqrt{1-(y / c)^{2}}} w_2 \ dx \ dy \end{aligned}$$

to find

$$\begin{aligned} C_1'(z) = \frac{Q}{3 \pi b^4 c^4} (-3 c^4 b' c'+b^3 c \left( -2 b'^2+c c''-14 c'^2\right) + \\ b c^3 \left( -14 b'^2+c c''-2 c'^2\right) +b^4 \left( c b''-3 b' c'\right) +b^2 c^2 \left( c b''-6 b' c'\right) ), \end{aligned}$$

which must be integrated numerically to obtain $C_1(z)$. We then have a complete expression for the second-order pressure gradient, Eq. 38.

**The integral constraint assumption.** In our solution, we have assumed that the 0th order flux $Q_0$ dominates the total flux Q, such that the second order flux $Q_2$ is negligible:

66 $$\begin{aligned} Q_0 = 4 \int _{0}^{c} \int _{0}^{b \sqrt{1-(y / c)^{2}}} w_0 \ dx \ dy \approx Q \end{aligned}$$

and

67 $$\begin{aligned} Q_2 = 4 \int _{0}^{c} \int _{0}^{b \sqrt{1-(y / c)^{2}}} w_2 \ dx \ dy \approx 0. \end{aligned}$$

We can justify that $Q_2 \ll Q_0$ by referring to Equation 37. In this equation, all of the terms are of equal order, so comparing the 2nd and 3rd terms

68 $$\begin{aligned} \left( \frac{\partial ^2}{\partial x^2} + \frac{\partial ^2}{\partial y^2}\right) w_2 \sim \frac{\partial ^2 w_0}{\partial z^2} \end{aligned}$$

and since $w_0 \sim Q_0$ and $w_2 \sim Q_2$,

69 $$\begin{aligned} \frac{Q_2}{b_0c_0} \sim \frac{Q_0}{L^2} \end{aligned}$$

and hence $Q_2/Q_0 \sim b_0c_0/L^2$, which is small for long, narrow ducts.

## Appendix 3: Calculation of the hydraulic resistance

The pressure gradient in the calculations of hydraulic resistance per unit length is calculated from the 3D simulations according to §3, which is a discretized approximation of

70 $$\begin{aligned} \frac{d}{dz} \left[ \frac{1}{A} \int _{A(z)} p \ dA \right] \end{aligned}$$

which is not equivalent to the quantity we calculate through lubrication theory (§ 4.1)

71 $$\begin{aligned} \left<\frac{\partial p}{\partial z}\right>= \frac{1}{A} \int _{A(z)} \frac{\partial p}{\partial z} \ dA \end{aligned}$$

since it does not take into account the rate at which the area changes axially. We can show, however, that these are approximately equal, that is,

72 $$\begin{aligned} \frac{d}{dz} \left[ \frac{1}{A} \int _{A(z)} p \ dA \right] \approx \frac{1}{A} \int _{A(z)} \frac{\partial p}{\partial z} \ dA \end{aligned}$$

if the amplitude of the change in area is relatively small. We demonstrate this with the circular duct, whose radius varies as *a*(*z*):

73 $$\begin{aligned} \frac{d}{dz} \left[ \frac{1}{A} \int _{A(z)} p \ dA \right]= & {} \frac{d}{dz}\left[ \frac{2}{a^2}\int _{0}^{a(z)} pr \ dr\right] \end{aligned}$$

74 $$\begin{aligned}= & {} 2 \left[ \frac{d}{dz}(a^{-2}) \int _0^{a(z)} p r \ dr + a^{-2} \frac{d}{dz}\int _0^{a(z)} pr \ dr\right] \end{aligned}$$

75 $$\begin{aligned}= & {} 2 \left[ -2a^{-3} a' \int _0^{a(z)} p r \ dr + a^{-2}\left( \int _0^{a(z)} \frac{\partial p}{\partial r} r \ dr + (p r)\vert _{a(z)} a'\right) \right] . \end{aligned}$$

The last term arises from the Leibniz rule, and takes into account $a'(z)$. The middle term is the quantity $\left<\partial p/\partial z\right>$.

76 $$\begin{aligned} \frac{d}{dz} \left[ \frac{1}{A} \int _{A(z)} p \ dA \right]= & {} \left<\frac{\partial p}{\partial z}\right>+ 2a' \left[ -2a^{-3} \int _0^{a(z)} p r \ dr + a^{-1} p\vert _{a(z)} \right] . \end{aligned}$$

If the radius *a* can be expressed as $a = a_0(1+\delta g(z))$, where $\delta \ll 1$ and *g*(*z*) is of O(1), then

77 $$\begin{aligned} \frac{d}{dz} \left[ \frac{1}{A} \int _{A(z)} p \ dA \right]\approx & {} \left<\frac{\partial p}{\partial z}\right>+ 2a_0 \delta g' \left[ -\frac{2(1-3\delta g+ O(\delta ^2))}{a_0^3} \int _0^{a(z)} p r \ dr + a_0^{-1}(1-\delta g + O(\delta ^2)) p\vert _{a(z)} \right] \end{aligned}$$

78 $$\begin{aligned}\approx & {} \left<\frac{\partial p}{\partial z}\right>+ O(\delta ). \end{aligned}$$

## References

[1] Iliff J, Wang M, Liao Y, Plogg B, Peng W, Gundersen G, Benveniste H, Vates G, Deane R, Goldman S, Nagelhus E, Nedergaard M. A paravascular pathway facilitates CSF flow through the brain parenchyma and the clearance of interstitial solutes, including amyloid $$\beta$$. Sci Transl Med. 2012;4(147):111–47. 10.1126/scitranslmed.3003748 PMC3551275 22896675

[2] Bohr T, Hjorth PG, Holst SC, Hrabětová S, Kiviniemi V, Lilius T, Lundgaard I, Mardal K-A, Martens EA, Mori Y, Nägerl UV, Nicholson C, Tannenbaum A, Thomas JH, Tithof J, Benveniste H, Iliff JJ, Kelley DH, Nedergaard M (2022). The glymphatic system current understanding and modeling. iScience..

[3] Kelley DH, Thomas JH (2023). Cerebrospinal fluid flow. Ann Rev Fluid Mechan.

[4] Asgari M, De Zélicourt D, Kurtcuoglu V (2015). How astrocyte networks may contribute to cerebral metabolite clearance. Sci Rep.

[5] Faghih MM, Sharp MK. Is bulk flow plausible in perivascular, paravascular and paravenous channels? Fluids Barriers CNS 2018;**15**(17) 10.1186/s12987-018-0103-8 PMC6003203 29903035

[6] Rey J, Sarntinoranont M (2018). Pulsatile flow drivers in brain parenchyma and perivascular spaces: a resistance network model study. Fluids Barriers CNS.

[7] Vinje V, Eklund A, Mardal K-A, Rognes ME, Støverud K-H (2020). Intracranial pressure elevation alters CSF clearance pathways. Fluids Barriers CNS.

[8] Tithof J, Boster KAS, Bork PAR, Nedergaard M, Thomas JH, Kelley DH (2022). A network model of glymphatic flow under different experimentally-motivated parametric scenarios. iScience.

[9] Boster KAS, Tithof J, Cook DD, Thomas JH, Kelley DH (2022). Sensitivity analysis on a network model of glymphatic flow. J Royal Soc Interf.

[10] Tithof J, Kelley DH, Mestre H, Nedergaard M, Thomas JH (2019). Hydraulic resistance of periarterial spaces in the brain. Fluids Barriers CNS.

[11] Vinje V, Bakker ENTP, Rognes ME (2021). Brain solute transport is more rapid in periarterial than perivenous spaces. Sci Rep.

[12] Raicevic N, Forer JM, Ladrón-de-Guevera A, Du T, Nedergaard M, Kelley DH, Boster K (2023). Sizes and shapes of periarterial spaces surrounding murine pial arteries. Fluids Barriers CNS.

[13] Min Rivas F, Liu J, Martell BC, Du T, Mestre H, Nedergaard M, Tithof J, Thomas JH, Kelley DH (2020). Surface periarterial spaces of the mouse brain are open, not porous. J Royal Soc Interf.

[14] Mestre H, Verma N, Greene TD, Lin LA, Ladron-de-Guevara A, Sweeney AM, Liu G, Thomas VK, Galloway CA, Bentley KLdM, Nedergaard M, Mehta RI (2022). Periarteriolar spaces modulate cerebrospinal fluid transport into brain and demonstrate altered morphology in aging and alzheimerÕs disease. Nature Commun.

[15] Boster KAS, Cai S, Ladrón-de-Guevara A, Sun J, Zheng X, Du T, Thomas JH, Nedergaard M, Karniadakis GE, Kelley DH (2023). Artificial intelligence velocimetry reveals in vivo flow rates, pressure gradients, and shear stresses in murine perivascular flows. Proc Natl Acad Sci.

[16] White FM (2006). Viscous Fluid Flow.

[17] Thomas JH (2019). Fluid dynamics of cerebrospinal fluid flow in perivascular spaces. J Royal Soc Interf.

[18] Wild R, Pedley T, Riley D (1977). Viscous flow in collapsible tubes of slowly varying elliptical cross-section. J Fluid Mechan.

[19] Tavakol B, Froehlicher G, Holmes DP, Stone HA (2017). Extended lubrication theory: improved estimates of flow in channels with variable geometry. Proc Royal Soc Math Phys Eng Sci.

[20] Housiadas KD, Tsangaris C (2022). High-order lubrication theory in channels and tubes with variable geometry. Acta Mechan.

[21] Bahrami M, Michael Yovanovich M, Richard Culham J (2007). A novel solution for pressure drop in singly connected microchannels of arbitrary cross-section. Int J Heat Mass Trans.

[22] Mestre H, Tithof J, Du T, Song W, Peng W, Sweeney A, Olveda G, Thomas J, Nedergaard M, Kelley D (2018). Flow of cerebrospinal fluid is driven by arterial pulsations and is reduced in hypertension. Nat Commun.

[23] White CM, Appleton EV (1929). Streamline flow through curved pipes. Proc Royal Soc London Series Contain Papers Math Phys Character.

